# “Our City Will Be the First to Hold Both Summer and Winter Olympics”: A comparative analysis of how media coverage and public opinion were framed on social media in the lead up to the Beijing 2022 winter Olympic games

**DOI:** 10.3389/fpsyg.2023.1125522

**Published:** 2023-03-01

**Authors:** Zesheng Yang, Yang Ruan, Jianing Zhang

**Affiliations:** ^1^Department of Audiovisual Communication and Advertising, Autonomous University of Barcelona, Barcelona, Spain; ^2^Independent Researcher, Zhengzhou, China; ^3^Faculty of Hospitality and Tourism Management, Macau University of Science and Technology, Taipa, Macau SAR, China

**Keywords:** Beijing 2022 winter Olympic games, media coverage, public opinion, sentiment analysis, social media

## Abstract

**Introduction:**

Beijing is the first city to host both the Summer and Winter Olympic Games. Mega sporting events such as the Olympic Games, which attract mass audiences, benefit greatly from social media. This article examines how the news coverage and public opinion about the Beijing 2022 were articulated on social media in the lead up to the Beijing 2022.

**Method:**

We employed computational content analysis to examine 9,439 individual posts and 450 official media posts that appeared before the Beijing 2022 Olympics. We also used ROSTCM6 to investigate the sentiment of official media and public opinion toward Beijing 2022.

**Results:**

The results of this study reveal that members of public are more inclined to highlight certain aspects of Beijing 2022 based on their individual perspectives. Official media, whose work generally aligns with the government’s interests. Through a sentiment analysis of these posts, we found strongly positive attitudes concerning Beijing 2022 among the Chinese public and the media.

**Discussion:**

Our results provide ample evidence of an overall relative convergence of positions between public opinion and news coverage about the Beijing 2022, despite their divergences. This study indicates that social media presents itself as a space for broader public statements, and empowers ordinary people to discuss China’s social issues of concern. Meanwhile, official media represents the government’s position, strategically framing Beijing 2022 as a landmark event in the new era of China.

## Introduction

By combining narratives involving both public issues and people’s private lives (Moreau et al., 2021), social media have opened up opportunities for individuals to engage with public and social issues (van Laer and van Aelst, 2010; Lee and Kwak, 2012). Through such narratives, people frame and reframe these debates (Meraz and Papacharissi, 2013). Social media not only disseminate individuals’ posts and perceptions; they also provide a platform for media organizations. Over the past decade, most traditional media outlets have expanded their influence through social media, ensuring that they have a strong online presence alongside their print publications (Obrien, 2011; Hong, 2012; Hiers, 2014).

Mega sporting events such as the Olympic Games, which attract mass audiences, benefit greatly from social media (Tang and Cooper, 2018). The 2016 Rio Olympic Games were called the ‘most social Olympics’ (Lui, 2016). With social media platforms becoming closely linked to the Olympics, the Olympic Games have entered an era known as ‘Socialympics’ (Liu, 2016). Researchers have argued that the use of social media during the Olympic Games has redefined the public domain or once again highlighted the value of sharing information online, with a lasting impact on society (AkhtPar, 2016; Girginova, 2016). In addition to promoting the Summer Olympics, digital technology and social media have been instrumental in establishing the Winter Olympics as a global mega sporting event too (Ramon et al., 2020).

During the bidding process for the 2022 Winter Olympics, at least five proposed cities backed out following voter referendums or public polls. Only Beijing, China, and Almaty, Kazakhstan were the only remaining candidate cities. Although voter referendums and public polls are not popular in China, in China’s media ecology, social media is considered to effectively reflect public opinion, while mainstream media is treated as the official mouthpiece (Stockmann and Luo, 2017; Li et al., 2019). Research on media reporting and social media content relating to sports is particularly valuable. As scholars noted that work comparing sports coverage in traditional media with discussions of sports on social media is important because it allows researchers to identify crucial differences in how the two forms of media report sporting events (Billings et al., 2015). Today, however, communications generated by the news media and general public coexist on interactive online platforms (Neubaum and Krämer, 2017). Treating ‘social media’ as a homogenous whole, encompassing both personal and news media content, cannot fully represent the reality of sports coverage. It is therefore necessary to further distinguish how media coverage and public opinion frame the mega sporting event on social media, and to compare specific topics presented by media outlets and public opinion. What is more, few researchers have studied how media frame and publics discuss mega sporting events before the competitions. Accordingly, this study compares the frames implicit in the media coverage of and public opinion concerning Beijing 2022 on Chinese social media during the one-year-long countdown to the event, as well as the sentiments expressed in relevant social media posts.

## Literature review

### Framing theory

A popular approach in political and communication studies, framing theory attends to media’s capacity to influence how a given audience understands an issue. Goffman (1974) initially put framing theory forward in his book *Frame Analysis*, in which he suggests that framing occurs when an individual stresses a certain relevant aspect to define a situation. Entman (1993) carries this forward into an analysis of media framing, arguing that news agencies discover the potential causes and possible outcomes of a given issue, make moral judgements and cover the topic accordingly. Framing theory shows how highlighting and selecting specific aspects of an issue can augment framing effects, thereby making these aspects more prominent in a communication environment (Chong and Druckman, 2007; Entman, 2007).

A number of sports communication researchers have used framing theory to study how mass media and social media understand the Winter Olympic Games. Billings and Eastman (2003) examined how identities were framed in broadcasters’ announcements of the 2002 Winter Olympics. Misener (2013) analyzed how the legacy of the 2010 Vancouver Winter Paralympic Games was framed in the media. Oh et al. (2020) investigated the media framing of the unified Korean Olympic women’s ice hockey team in newspaper coverage and social media commentary. What is more, scholars have examined the framing of the Olympics with regard to social and political issues (Seippel et al., 2016; Pan and Lawal, 2017) such as environmental degradation (Yoon and Wilson, 2019), public protest (van Luijk and Frisby, 2012).

### Mega sporting events in China

In China, mega sporting events have long been seen as an important way of strengthening national identity, stirring up patriotism among its citizens, enhancing its international standing, and projecting an image of unity and modernity abroad (Chen, 2012; Chu, 2018). After the 2008 Beijing Olympic Games, which successfully altered China’s international image (Chung and Woo, 2011), China has continuously hosted the Asian Games, Summer Universiade, and Youth Olympic Games. The Chinese government’s next goal is to host a successful Winter Olympics.

Since the first Winter Olympics in 1924, the games have grown exponentially, becoming one of the most influential competitions in the world (Chappelet, 2002). In November 2013, the Chinese Olympic Committee formally nominated Beijing as a bidding city for the 2022 Winter Olympics. In 2015, after 2 years of preparation, Beijing was selected to host the XXIV Olympic Winter Games, becoming the first city to put on both the Summer and Winter Games. The Chinese government attaches great importance to the preparations for Beijing 2022. In March 2019, the State Council and Central Committee of the General Office of the Communist Party of China (CPC) issued a report, ‘Opinions on Developing Snow and Ice Sport as an Opportunity for Beijing 2022’ (Xinhua News Agency, 2019), which further emphasized the significance of the event. Additionally, Xi Jinping has given the Beijing Winter Olympics a distinctly personal touch. According to media reports, Xi is an avid sports fan and enjoys winter sports in particular (Danson, 2019; Huaxia, 2020). The 2022 Winter Olympics therefore promises to be among the landmark events of his presidency.

### Using Weibo data to study media coverage and public opinion

Launched by Sina Corporation in August 2009, Weibo is the only vibrant micro-blogging platform in China, with more than 553 million users in the first quarter of 2021 (Guancha Syndicate, 2021). Although Weibo began as a Chinese equivalent of Twitter, it has since incorporated several features that resemble Facebook. Weibo offers great visibility and extensive news coverage. Accordingly, it is the most influential platform in China for discussing popular news and public issues (Huang and Sun, 2014; Rauchfleisch and Schäfer, 2015).

Given the growing importance of social media in media coverage and the formation of public opinion, in 2014 the CPC leadership released a memorandum promoting media convergence and the digitalization of mainstream media. It should be pointed out that in China mainstream news organizations are certified by the government, for they are crucial for consolidating the CPC’s political power (Daniela and Mary, 2011; Qin et al., 2018). After 2014, mainstream media began establishing a large number of Weibo accounts to publish news and attract readers. According to the ‘Weibo User Development Report’ of 2020, it had 38,000 verified mainstream media accounts by the end of the year (Weibo, 2021). In the light of the mainstream media’s great influence on Weibo, we set out to establish which frames were prominent in mainstream news media coverage of Beijing 2022 on Weibo during the pre-Olympic period.

In recent years, researchers have become increasingly interested in using social media as a meter of public opinion. Despite there being some drawbacks to deriving public opinion from social media, many researchers have demonstrated that social media data are a strong indicator of how publics perceived certain issues (Anstead and O’Loughlin, 2015; Choi and Lee, 2017; Neubaum and Krämer, 2017). Some scholars have extracted Weibo data to explore public opinion in China. Despite the existence of censorship and deletion measures on Chinese social media (Rauchfleisch and Schäfer, 2015), they argue, the Chinese government allows online debate on public affairs, permitting users to respond to government policies as long as online debates do not catalyze collective action (King et al., 2017). In comparing several Chinese social media, Stockmann and Luo (2017) concluded that Weibo may have the greatest potential to facilitate online discourse on a particular topic. Another group of researchers (Li et al., 2019) analyzed Weibo data to summarize public opinion regarding public transportation problems in China and proposed improvement measures. Still another (Han et al., 2020) examined Chinese public opinion during the first stages of COVID-19, finding that Weibo texts were an accurate and feasible means through which to do so. Very few studies have used Weibo data to study public opinions about mega sporting events, however. Accordingly, alongside our framing analysis of mainstream media, we aim to identify prominent frames in public discussions of Beijing 2022 on Weibo during the run-up to the Olympics.

### Sentiment analysis

Sentiment analysis (which attends to people’s perspectives, evaluations, attitudes, and emotions) is among the most active areas of research in natural language processing. Having expanded beyond computer science to the social sciences, this research method is now essential to understanding public opinion. Social media has made it possible for millions of people to share their emotions and attitudes on online social networks, leading to an explosion in the volume of relevant data. Using sentiment analysis to examine these data allows researchers to learn about public attitudes and social trends (Di et al., 2014).

Analyzing sentiments on Weibo has become increasingly important in academic study. A number of scholars have developed research methods to examine sentiments in Weibo, including using deep neural networks (Wan, 2019) and latent Dirichlet models (Yonggan et al., 2016). Weibo posts have also been used to examine public sentiments regarding public affairs such as Hong Kong’s ‘Occupy Central’ movement (Luo and Zhai, 2017); stock market fluctuations (Chen et al., 2016); how incidents are monitored following disasters (Bai and Yu, 2016); and debates over traditional Chinese medicine (Shen et al., 2015). Few studies, however, analyze the sentiments surrounding mega sporting events. In view of this, we examine the sentiments expressed concerning Beijing 2022 in the pre-Olympic period.

## Method

### Data collection

To understand the frames embedded in news media and public posts, we collected content posted on Weibo in the year leading up to the opening of Beijing 2022. Given that the one-year countdown to the games began on 4 February 2021, we gathered data between 4 January and 28 February 2021. Many milestone events and celebrations were held to usher in Beijing 2022 in and around these 2 months. For instance, Beijing 2022 sports pictograms were released; the games’ competition venues were all completed and unveiled; the torch was released at a celebration marking the beginning of the one-year countdown; and test events for domestic athletes was held. These events received extensive media coverage and public attention, producing an abundance of social media data.

We employed a web crawler to collect posts on Weibo. Specifically, data was collected in the following steps. First, to identify posts, we conducted searches in Chinese for the keywords ‘Beijing 2022 Winter Olympic Games’ (北京2022年冬奥会) and ‘Beijing Winter Olympic Games’ (北京冬奥会). The posts’ content, account name, release time, account type, and other publicly visible information were collected. Second, to accurately represent the public discourse, posts from government agencies, enterprises, news media, and other organizational users were excluded. 9,439 posts from individual accounts were retained as the sample. Third, we conducted searches on Weibo for news media accounts that met the following three criteria: Chinese central government-controlled media; mainstream media in Beijing, the games’ host city; and Chinese professional sports media. Our list was filtered and supplemented by ten professional sport journalists with at least 5 years’ experience in China. Finally, we chose 23 influential news media accounts from which to collect content, and reused the keywords used in the first step to gather relevant content.^1^ This process eventually produced a sample of 450 Weibo posts.

### Data analysis

#### Topic modelling

To investigate the topics appearing in public discussions and news reports, we conducted topic modelling: a computational, unsupervised method of effectively extracting patterns of meaning from large numbers of unstructured posts (Blei, 2012). It is often used to identify not only topics and frames, but also agendas in large social media datasets (Guo et al., 2016; Walter and Ophir, 2019; Li et al., 2020). Put simply, topic modelling enables users to organize and summarize large volumes of documents that would be impossible to annotate manually. Before analysis, we cleaned up the data by following standard pre-processing steps developed in previous studies (removing all stop words, punctuation marks, numbers, and non-lexical characters). Later, we segmented the text into words using the Jieba package in Python. Adopting the Latent Dirichlet Allocation (LDA) as a topic-modelling algorithm, we used it to cluster words from personal documents into different topics. Based on the probability of keyword co-occurrence, each topic was fed through an extracted cluster of keywords (Wang et al., 2021). Considering both the relatively low value of perplexity and dearth of topics, we identified 10 topics for individual accounts and 14 topics for media accounts after several attempts at completing this step of the process.

#### Manual coding of frames

We then read all the topics and high-frequency words. By evaluating the extent to which the topics were exclusive of one another and merging the similar topics, we manually coded the ten extracted topics for individual accounts into four frames (as shown in Table 1): entertainment stars, public attitude, preparation work, and Olympic partners. Using the same approach, we clustered the 14 topics for media organization’s accounts into four frames: preparatory work, political issues, COVID-19 pandemic, and media attitudes (Table 2). This step is in line with the proposition that LDA-generated topics can be regrouped or recoded into meaningful, condensed frames based on conceptual similarities (Nelson, 2020), which has been widely applied in the previous literature (Maier et al., 2018; Li et al., 2020).

**Table 1:** List of extracted frames and keywords of individual accounts.

Frame	Topic	*n* (%)	High frequency words
Entertainment	0	642(6.80%)	Zhou Shen, Li Qin, ambassador, participation, send blessings
Stars	1	1,534(16.25%)	Xiao Zhan, You Changjing, Cool Guy, CCTV gala, new song
	2	583(6.18%)	Chen Linong, Yang Mi, Yang Zi, Meng Meiqi, culture
	3	881(9.33%)	Wang Jiaer, Yaochen, artist, sing, youth
Public attitude	4	1,291(13.68%)	encouragement, highlight, history, complete success
	5	718(7.60%)	joyous festival, happy get-together, power, anniversary, worldwide
	6	811(8.59%)	dream, cheer, congratulation, nation, blessing
Preparation work	7	939(9.95%)	Mascot, icon, ski jumping, snow, construction
	8	732(7.76%)	Zhang Jiakou, volunteer, high-speed train, ice-making
	9	786(8.32%)	Shou Gang, winter dream, ski resort, build, test
Olympic partners	10	522(5.53%)	Olympic, partner, China Unicom, Tsingtao, Sankeshu

**Table 2:** List of extracted frames and keywords in news media.

Frame	Topic	*n* (%)	High frequency words
Preparation Work	0	19(4.22%)	one-year countdown, qualification, athlete, ice-making, efficient
	1	35(7.78%)	Bing Dwen Dwen, Shougang, work progress, Bach, ice stadium
	2	46(10.22%)	competition, technology, Chongli, ice ribbon, torch
	3	38(8.44%)	construction, sprint, enthusiasm, all-out, domestic
Political issues	4	56(12.44%)	extreme sport, biathlon, challenge, attention, economic
	5	30(6.67%)	city of twice Olympics, general secretary, capital, Hebei, encouragement
	6	32(7.11%)	president, nation, Beijing 2022, ensure, do well
	7	31(6.89%)	Xi Jinping, perfect, Winter Olympic Games, champion, environmental protection
	8	15(3.33%)	regulation, professional, request, important speech, promotion
COVID-19	9	21(4.67%)	CEEC, IOC, communication, coordination, development
	10	25(5.56%)	epidemic, unity, preparation, join hands, investigate,
	11	24(5.33%)	COVID-19, Japan, Tokyo, collaborate, close to, hope
Media attitude	12	21(4.67%)	vaccine, solutions, orderly, criterion, civilisation
	13	24(5.33%)	strong, powerful country, innovation, citizen, speed
	14	33(7.33%)	confidence, successfully, cohesion, spirit, whole society

#### Sentiment analysis

**Figure 1:**
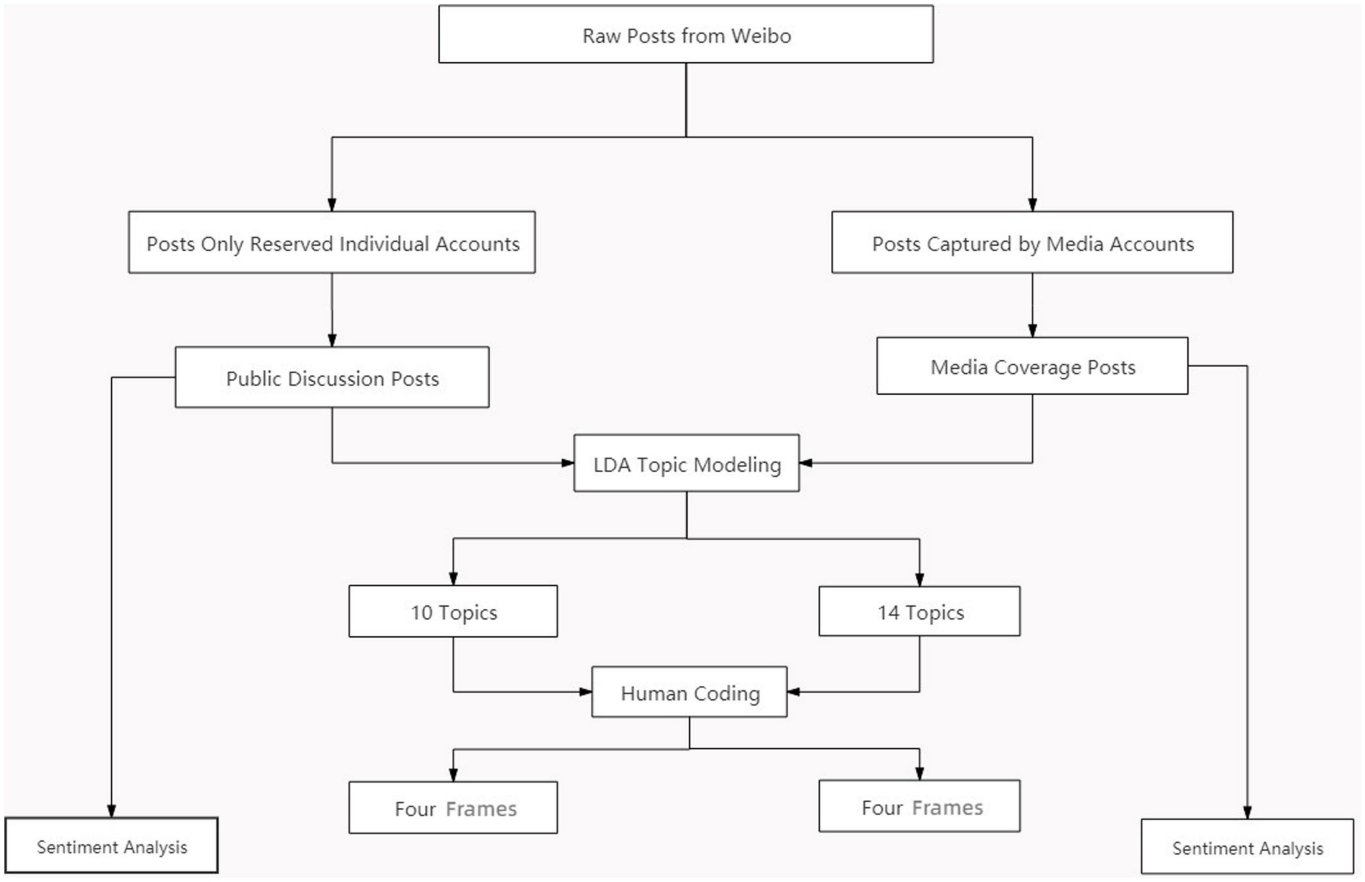
The flow of data processing.

Sentiment analysis is a fundamental text-analysis technique. It works by classifying the polarity of a text and identifying positive and negative sentiments (Wilson et al., 2005). For this study, we adopted the Rost Content Mining 6 (ROSTCM6) system, which captures sentimental expressions and their patterns in the Chinese language. The application assigns polarity scores for each text body as positive, negative, or neutral by comparing derived patterns and pre-examined text structures. Whereas positive sentiments include attitudes of support, consensus, or trust, negative sentiments express opposition or rejection (Kiritchenko et al., 2014). If a document contains more positive scores than negative and neutral scores, it is deemed as positive, and vice versa (Tan and Zhang, 2008). In ROSTCM6, neutral sentiment is expressed as 0. It serves as the dividing line between positive and negative sentiments, which are expressed as numbers above and below 0, respectively. Positive and negative sentiments were graded on a scale of 10 from low, through moderate, to high (Figure 1).

## Results

### Frames in public opinion

Four frames emerged from the public opinion expressed concerning Beijing 2022. The first frame, which was articulated in most of the posts (*n* = 3,640, 38.56%) we analyzed, referred to Beijing 2022 promotional activities involving entertainment stars. Many pop stars attended the numerous events held to celebrate there being just 1 year left to the games. The countdown coincided with the Chinese Lunar New Year, the most important traditional festival in China. Several singers performed Olympic-themed songs on China’s most-watched television program, China Central Television (CCTV) Spring Festival Gala. The show garnered huge popularity, as one of the individual Weibo posts we analyzed makes clear:

Yang Mi, Jin Chen, and Li Qin are really good-looking people! Actors @Jin Chen participated for the first time in the 2021 CCTV Spring Festival Gala. Elegant and moving, her voice is gripping, with a song entitled ‘Burning Snowflake’, for all the athletes cheering for 2022 Beijing!

The second frame expressed by the public (n = 2,820, 29.87%) is attitude towards Beijing 2022. Most Chinese ‘netizens’ eagerly anticipated that games, using words such as ‘encouragement’, ‘reunion’, ‘dreams’, ‘celebration’, and ‘complete success’ to express their enthusiasm. One post reads: ‘Looking forward to Beijing 2022, for dreams and faith! Fight for honor, cheer for yourself! We await Beijing 2022 together!’

Some individual users, however, expressed critical views of Beijing 2022. In their opinion, winter sports are not popular in China, especially in the south, and ensuring that the entire nation benefits from Beijing 2022 will be a challenge. A few users argued that China no longer needed to hold sporting events to demonstrate national strength and enhance its status; it would be better to spend those funds on improving people’s livelihoods instead.

The third frame consisted of public discussions (*n* = 2,457, 26.03%) about the preparatory work for Beijing 2022. The public mostly focused on the milestones leading up to Beijing 2022 that were relevant to them personally, such as the recruitment of volunteers and opening of the Beijing-Zhangjiakou high-speed rail line. For instance, in one post a user wrote: ‘I want to be a volunteer for Beijing 2022. I hope my dream comes true, wish me luck’.

Finally, many users (*n* = 522, 5.53%) also discussed the Olympic partners of Beijing 2022. For many companies, social media have become an important platform on which to promote their products and enhance their brand value (Ashley and Tracy, 2015). Olympic partners took advantage of the beginning of the 1-year countdown to Beijing 2022 to launch marketing campaigns that attracted the attention of many consumers.

### Frames in news media

Four frames were extracted from the 14 news media topics: preparation work, political issues, COVID-19, and media attitudes. The first frame (*n* = 194, 43.11%) was preparation work for Beijing 2022. Although this frame was present in posts by individual accounts too, they expressed different concerns about the events in question. Media outlets focused on Beijing 2022 milestone events and the construction of basic facilities, as represented in the following quote: ‘Tonight, the torch of Beijing 2022–‘Flying’ was officially released. Its overall design echoes that of the torch tower at the opening of the Beijing 2008 Olympic Games’.

Moreover, words such as ‘sprint’, ‘enthusiasm’, and ‘go all out’ frequently appeared in this frame, reflecting the media’s confidence in the success of Beijing 2022.

The second frame (*n* = 129, 28.67%) that emerged from the data was political issues. In this frame, words such as ‘Xi Jinping’, ‘president’, ‘General Secretary’, ‘regulations’, ‘Central and Eastern Europe’, and ‘coordination’, were used to highlight the importance of the Chinese government and its diplomatic activities in relation to Beijing 2022. During the key stage of the preparations for Beijing 2022—between 18 and 20 January 2021—Xi Jinping, general secretary of the CPC, visited competition venues in Beijing and Hebei. The purpose of his visit was to assess how preparations were going. This news was extensively reported. Consider the following Weibo post:

From January 18 to 19, CPC General Secretary Xi Jinping inspected the three competition areas in Beijing and Hebei to cheer for Beijing 2022. Xi’s concern for Beijing 2022 reveals his profound thinking and overall strategic planning for a strong sporting nation and a healthy China.

The third frame (*n* = 70, 15.56%) was COVID-19; relevant words included ‘coronavirus’, ‘pandemic’, and ‘vaccine’. This frame encompassed three kinds of content. The first is content related to how Beijing 2022 was being prepared during a pandemic. Additionally, the media reported on the National Health Commission’s response to the measures being implemented to control the pandemic in the context of Beijing 2022. The cooperation between China and international organizations (especially the International Olympic Committee) in fighting the virus also attracted the media’s attention. The president of the International Olympic Committee, Thomas Bach, said that ‘China has performed well in responding to the COVID-19 pandemic, and the International Olympic Committee is full of confidence in the success of Beijing 2022’.

The fourth frame (*n* = 57, 12.66%) was media attitudes. The news media’s attitudes towards Beijing 2022 are most prominently articulated through editorials, which are particularly expansive in terms of expression. The media revealed its attitude by using phrases such as ‘a powerful country’, ‘smooth development’, ‘the whole world’, and ‘full of confidence’. As the literature points out, Chinese media is largely a mouthpiece of the government. It is therefore logical that their coverage is consistent with the government’s stance on major issues.

In summary, a comparison of the frames that emerged from individual users and news media accounts revealed that both groups discussed the preparations for Beijing 2022 and milestones that led to the opening of the games. Among individual accounts, Weibo users were more inclined to discuss content related to entertainment stars. In contrast, entertainment stars rarely appeared in the news media. The vast majority of media reports focused on preparation work and political content, topics that were not significant in personal accounts.

### Sentiment analysis

In undertaking a sentiment analysis of 9,439 personal and 450 news organization Weibo posts, we found that the majority of posts in both categories expressed positive sentiments. Whereas 70.20% of personal accounts were positive, the figure is 84.66% for media accounts. The emotions articulated in media accounts (which represent official ideologies) were more positive, as compared with personal accounts (Table 3).

**Table 3:** Sentiments of the public and media concerning Beijing 2022.

	Positive (%)			Neutral (%)	Negative (%)			Total
	Low	Moderate	High		Low	Moderate	High	
Public	2,755(29.19)	1907(20.20)	1964(20.81)	1,627(17.24)	594(6.29)	189 (2.00)	403 (4.27)	9,439
Media	127(28.22)	73(16.22)	181(40.22)	43(9.56)	15(3.33)	8(1.78)	3(0.67)	450

The positive sentiments found in personal accounts arose from the fact that a large number of fans expressed support for the popular idols who participated in promotion campaigns for Beijing 2022. The news media’s high degree of positivity was indicated by its coverage of how national leaders’ approved the preparations for Beijing 2022. Additionally, news about Chinese athletes’ hard work and preparations for the games also accounted for a large proportion of positive statements. Of the three sentiments, neutrality was the least prominent. Whether in public posts or new media, this emotion was primarily expressed in factually supported statements.

The negative sentiments expressed in the news media had to do with some winter sports being postponed or cancelled. Some of these competitions were warm-up matches to be held ahead of Beijing 2022 or qualifying competitions for the Winter Olympics. The postponement of the Tokyo 2020 Olympic Games also raised concerns about whether Beijing 2022 could be conducted on schedule. The negative sentiments expressed by the public had more diverse sources. Some negative statements suggested that Beijing 2022 may not positively impact people’s lives and could disrupt the natural environment. Individual users also responded negatively to calls to boycott Beijing 2022 in some Western countries.

## Discussion

### Media coverage frames vs. public opinion frames

This research on how Beijing 2022 was framed in media and public opinion on Weibo has produced findings that enhance our understanding of the Chinese people and media’s opinions of mega sporting events. Interestingly, our results revealed that public discussion regarding Beijing 2022 differs significantly from how it was covered in the media. The extent to which the news media set the agenda for public opinion seems to be waning.

Entertainment stars have become the most prominent topic of discussion among personal accounts. A number of well-known Chinese entertainment stars – including Xiao Zhan, Wang Jiaer, Yang Mi, Yang Zi, and Meng Meiqi – were mentioned many times. Promotional campaigns mounted by these pop stars, such as the Spring Festival Gala and the release of Winter Olympic songs, also received great attention from admirers. This study also corroborates Figuera and Nieto (2018) findings, which suggest that content related to entertainers and fans accounted for a large percentage of social media engagement during the Pyeongchang 2018 Winter Olympics.

The most discussed topics on news media were political issues. This finding makes sense because the Chinese media, as the mouthpiece of the CPC, is a tool of regime legitimization and effective rule (Daniela and Mary, 2011; Qin et al., 2018). When it came to political issues, the terms ‘Xi Jin Ping’, ‘General Secretary’, and ‘Chairman’ were used frequently. Xi Jinping regards Beijing 2022 as a landmark event, which endows Beijing 2022 with his personal characteristics. This is in line with his media image as an avid sport enthusiast (Danson, 2019; Huaxia, 2020). Words such as ‘strong’, ‘powerful’, ‘people’, and ‘full of confidence’ were frequently mentioned among media outlets. Our results confirm those of previous studies to the effect that China uses international sporting events to enhance its national identity and establish its authority (Chen, 2012; Chu, 2018). The COVID-19 pandemic is another focus of attention in the media accounts. The word ‘vaccines’ appear frequently in media reports, indicating that China has adopted an effective plan to combat the pandemic and ensure that Beijing 2022 proceeds smoothly.

Although we found that the public and the media’s positions were relatively consonant when it came the preparation work, the specific issues of concern are different. Individual accounts tend to focus more on personal events, such as volunteer recruitment activities. The official media continued the consistent grand narrative for mega-event, covering topics such as test games, the production of ice and snow in venues, and infrastructure construction, but lack of coverage of how Beijing 2022 affects individuals. Such a reporting strategy formed a separation between official propaganda and public opinion on the affairs of the Beijing 2022. It is difficult for ordinary people to resonate with official reports. Additionally, the finding of personal accounts critical of the Beijing 2022 echoed previous research (Rauchfleisch and Schäfer, 2015; King et al., 2017), providing further evidence that the Chinese government allows public criticism on certain issues on social media, such as sports.

### Sentiment for Beijing 2022

This study reveals sentiments concerning Beijing 2022 among individuals and news media. The Chinese public and media have generally expressed positive attitudes towards the event. The results indicate that Chinese people are still very enthusiastic about mega sporting events and hope to perpetuate the fame of the 2008 Beijing Olympics. The support for Beijing 2022 in China contrasts with the opposition to mega sporting events in other countries. Some Western cities even withdrew their bids to host the Olympic Games following strong public opposition (Kassens-Noor and Lauermann, 2018; Paulsson and Alm, 2020). That said, it is important to acknowledge the negative sentiments expressed in the data. These concerned a range of public affairs, including the waste of public funds, damage to the environment, and the spread of COVID-19.

## Conclusion

Against the backdrop of the COVID-19 pandemic and many cities’ reluctance to bid for the Olympics, Weibo data concerning Beijing 2022 has generated numerous significant frames. Our results provide ample evidence of an overall relative convergence of positions between public opinion and news coverage about the Beijing 2022 Olympic Games, despite their divergences. This study indicates that social media presents itself as a space for broader public statements, and empowers ordinary people to discuss China’s social issues of concern. Meanwhile, news media represents the government’s position, strategically framing Beijing 2022 as a landmark event aimed to increase the rule of the CPC. This indicates the influence of China’s political ideology and media ecology.

This study also has a few limitations. Firstly, public opinions expressed on social media could be contaminated by bot accounts and hired internet commentators (King et al., 2017). Identifying and eliminating these distorting factors requires rigorous and sophisticated data-cleaning processes. Secondly, we only collected data relating to the year leading up to Beijing 2022. We cannot examine the dynamic relationship of official media and individual media over a longer period of time, and how official media responds to negative sentiment in individual media. Future research might investigate how the Chinese public and media perceived Beijing 2022 over a longer duration.

## Data availability statement

The original contributions presented in the study are included in the article/supplementary material, further inquiries can be directed to the corresponding author.

## Author contributions

ZY contributes to data collection, data analysis, and writing the original manuscript. YR helps to review and check the final revision. JZ contributes to editing the format of the manuscript. All authors contributed to the article and approved the submitted version.

## Conflict of interest

The authors declare that the research was conducted in the absence of any commercial or financial relationships that could be construed as a potential conflict of interest.

## Publisher’s note

All claims expressed in this article are solely those of the authors and do not necessarily represent those of their affiliated organizations, or those of the publisher, the editors and the reviewers. Any product that may be evaluated in this article, or claim that may be made by its manufacturer, is not guaranteed or endorsed by the publisher.

## Footnotes

^1^The news media accounts are: 人民日报 (People’s Daily), 新华社 (Xinhua News Agency)，人民日报体育 (People’s Daily-Sport)，新华体育 (Xinhua News-Sport)，央视体育 (CCTV-Sport)，人民体育 (People’s -Sport)，新华网 (Xinhua Net)，中央人民广播电台 (China National Radio)，央视新闻 (CCTV-News)，BTV冬奥纪实 (BTV Documentary)，环球时报 (Global Times)，北京日报 (Beijing Daily)，河北日报(Hebei Daily)，河北广播电视台 (Hebei Radio and TV Station)，中国新闻网 (China News)，体坛周报 (Titan Sport)，中国青年报 (China Youth Daily)，中国体育报(China Sport Daily)，澎湃新闻 (The Paper)，光明日报 (Guangming Daily)，科技日报 (Science and Technology Daily)，南方都市报 (Southern Metropolis Daily), and 北京晚报 (Beijing Evening).
